# Place Cell Rate Remapping by CA3 Recurrent Collaterals

**DOI:** 10.1371/journal.pcbi.1003648

**Published:** 2014-06-05

**Authors:** Trygve Solstad, Hosam N. Yousif, Terrence J. Sejnowski

**Affiliations:** 1 Howard Hughes Medical Institute, Computational Neurobiology Laboratory, Salk Institute for Biological Studies, La Jolla, California, United States of America; 2 Kavli Institute for Systems Neuroscience and Centre for Neural Computation, Norwegian University of Science and Technology, MTFS, Trondheim, Norway; 3 Department of Physics, University of California at San Diego, La Jolla, California, United States of America; 4 Division of Biological Sciences, University of California at San Diego, La Jolla, California, United States of America; Indiana University, United States of America

## Abstract

Episodic-like memory is thought to be supported by attractor dynamics in the hippocampus. A possible neural substrate for this memory mechanism is rate remapping, in which the spatial map of place cells encodes contextual information through firing rate variability. To test whether memories are stored as multimodal attractors in populations of place cells, recent experiments morphed one familiar context into another while observing the responses of CA3 cell ensembles. Average population activity in CA3 was reported to transition gradually rather than abruptly from one familiar context to the next, suggesting a lack of attractive forces associated with the two stored representations. On the other hand, individual CA3 cells showed a mix of gradual and abrupt transitions at different points along the morph sequence, and some displayed hysteresis which is a signature of attractor dynamics. To understand whether these seemingly conflicting results are commensurate with attractor network theory, we developed a neural network model of the CA3 with attractors for both position and discrete contexts. We found that for memories stored in overlapping neural ensembles within a single spatial map, position-dependent context attractors made transitions at different points along the morph sequence. Smooth transition curves arose from averaging across the population, while a heterogeneous set of responses was observed on the single unit level. In contrast, orthogonal memories led to abrupt and coherent transitions on both population and single unit levels as experimentally observed when remapping between two independent spatial maps. Strong recurrent feedback entailed a hysteretic effect on the network which diminished with the amount of overlap in the stored memories. These results suggest that context-dependent memory can be supported by overlapping local attractors within a spatial map of CA3 place cells. Similar mechanisms for context-dependent memory may also be found in other regions of the cerebral cortex.

## Author Summary

The activity of ‘place cells’ in hippocampal area CA3 systematically changes as a function of the animal's position in an arena as well as contextual variables like the color or shape of enclosing walls. Large changes to the local environment, e.g. moving the animal to a different room, can induce a complete reorganization of place-cell firing locations. Such ‘global remapping’ reveals that memory for different environments is encoded as separate spatial maps. Smaller changes to features within an environment can induce a modulation of place cell firing rates without affecting their firing locations. This kind of ‘rate remapping’ is still poorly understood. In this paper we describe a computational model in which discrete memories for contextual features were stored locally within a spatial map of place cells. This network structure supports retrieval of both positional and contextual information from an arbitrary cue, as required by an episodic memory structure. The activity of the network qualitatively matches empirical data from rate remapping experiments, both on the population level and the level of single place cells. The results support the idea that CA3 rate remapping reflects content-addressable memories stored as multimodal attractor states in the hippocampus.

## Introduction

The rodent hippocampus forms a neural representation of the local environment using a dual rate and position code: Each pyramidal neuron is active when an animal is located within a distinct location in space [1], and its mean firing rate within this location varies with contextual features like the color or shape of enclosing walls [2]. These two coding schemes can be distinguished by observing how the network 'remaps' in response to changes in the local environment. Exposing the animal to a physically different space has been shown to induce global remapping, in which both location and firing rate of place cells take entirely new values [2]–[5]. Different environments therefore appear to be encoded by separate spatial maps [6]. In contrast, manipulating contextual features like the color or shape of surrounding walls [2], odors [7], or task [8] within the same space can elicit substantial changes in firing rates while the location of place fields is unaffected. Such ‘rate remapping’ can affect behavioral decisions [8], and appears to reflect the presence of multiple context-dependent memories stored within a single spatial map.

According to current attractor network theory (e.g. [9]–[11]), discrete attractor dynamics for contextual memory is expected to manifest as an abrupt shift in the neural representation as one context is morphed into the next, as is the case for global remapping. Contrary to this prediction, a gradual transition in the population activity of CA3 place cells was observed as a familiar square arena was morphed into a familiar circular arena in six steps following training in a rate remapping paradigm [12]. These results imply that place cell firing rates are not dictated by network-wide attractor dynamics of the Hopfield type [5], [12].

On the other hand, both gradual and abrupt transitions were observed on the single neuron level, and in CA3 the transition points of single neuron firing rates also depended on the direction of morphing, a form of hysteresis characteristic of nonlinear systems [13]. We hypothesized that discrete attractors embedded in the CA3 network might give rise to different dynamics than those of Hopfield type networks due to effects of spatially dependent connections between place cells. Building on previous work on combining discrete and continuous attractor networks [14]–[16], we demonstrate here that a network model in which local attractors for contextual features are embedded in a continuous attractor for spatial position can account for the apparent conflict in the evidence for attractor dynamics in experimental data on rate remapping.

## Results

### Network model

**Figure 1:**
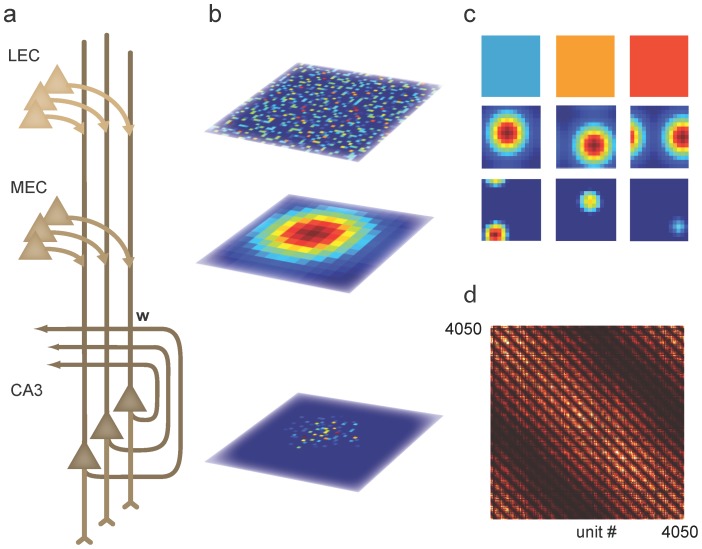
Combined continuous and discrete attractor model for episodic-like memory. ***a***, Network architecture. Each CA3 unit received dedicated LEC and MEC population inputs in addition to recurrent activity from other CA3 units connected through the weight matrix ***w***. ***b***, Network activity. **Top**: Each context was defined by a unique pattern of static LEC activity. Firing rate is color coded from dark blue (zero) to dark red (maximal). **Middle**: MEC activity defined a position within the environment and was identical between contexts. For each of the 225 positions eighteen place cells received identical spatial input. **Bottom**: Combining inputs from the MEC and LEC, the population activity in CA3 represents both the position of an animal within an environment and the currently active context by the particular pattern of firing rates within the active cell ensemble. ***c***, Single unit activity. **Top row**: Single LEC units had distinct but spatially homogeneous firing rates, shown as difference in color. **Middle row**: Single MEC units had broad Gaussian fields, representing the summed activity of several spatially modulated MEC neurons. **Bottom row**: Place field responses from units in the feedback model of CA3 had context modulated firing, affecting both peak firing rate and place field size. ***d***, The recurrent weight matrix had a spatial component (diagonal band) and a discrete, contextual component (discrete pattern within band). Periodic boundary conditions are visible as stripes in bottom left and top right corners. Colors denote zero weights in dark shades to maximal weights in bright shades.

To investigate the role of attractor dynamics in rate remapping we considered a recurrent neural network model of hippocampal area CA3 in which each position in the environment was represented by a group of CA3 units. External synaptic input from medial (MEC) and lateral (LEC) entorhinal cortices carried information about rat position and external sensory cues (context) respectively, in line with anatomical and physiological evidence (Figure 1a) [17], [18]. The activity of the *i*th hippocampal unit, *r_i_*, evolved according to 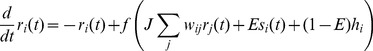
(1)where *s_i_(t)* and *h_i_* denote the synaptic input at time *t* to the *i*th hippocampal neuron from MEC and LEC respectively. *E = 0.8* is a constant determining the relative contribution of these two inputs. The parameter *J* determined the strength of recurrent feedback from stored memories.

Memories were stored using a Hebbian learning rule where the synaptic weight between the *i*th and *j*th hippocampal neuron, *w_ij_*, depended both on the Euclidean distance between their place field peak positions || *(x_i_ - x_j_, y_i_ - y_j_)* || *and* their peak firing rates (*ξ_i_^m^, ξ_j_^m^*) in each of *M* stored context memories (Figure 1d):
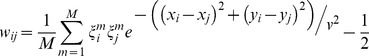
(2)where ν = *0.3* *L* defined the place field spatial scale in a square arena of side length *L = 75* *cm*.

Activity was passed through a non-linear activation function *f(u)* with divisive normalization [19]:
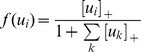
(3)Where [ ]_+_ denotes rectification.

Each LEC input, *h_i_,* was spatially homogeneous and independent of time, but had a distinct firing rate for each context, e.g. *h_i_ = ξ_i_^1^* for context A and *h_i_ = ξ_i_^2^* for context B (Figure 1c, top row; Methods). The number of overlapping units in rate vectors ***ξ*** *^1^* and ***ξ*** *^2^* for each position was denoted by the parameter *a*.

Each MEC input, *s_i_(t)* had a unimodal spatial profile (Figure 1c, middle row) that was identical for all contexts, approximating the sum of input from grid cells and other spatially modulated cells [20], [21]

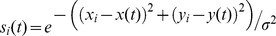
where (*x(t),y(t)*) denotes the position of the rat at time *t* in units of bins, (*x_i_,y_i_*) is the preferred firing location of the *i*th hippocampal neuron, and *σ = 0.3 L* denotes the width of the MEC input relative to the side length, *L = 75 cm*, of the arena. MEC input controlled the position of the hippocampal output without significantly affecting place field size.

Rat behavior was simulated by letting the peak of *s(t)* visit every position in the 15-by-15 bin environment once, following a smooth trajectory. The resulting place cells had single place fields with differing positions and firing rates (Figure 1c, bottom row). The identity of the active CA3 neurons encoded rat position as in standard continuous attractor models, whereas the specific pattern of firing rates within the active cell ensemble represented discrete contextual information stored at that particular location (Figure1b, bottom panel).

### Rate remapping

To investigate the effect of recurrent collaterals on rate remapping, we stored two contextual memories representing the square and circular enclosures of the rate remapping experiments in the synaptic weight matrix (Eq. 2). We focused our investigation on the effect of two parameters: (1) The strength of the recurrent feedback, *J*, and (2) the similarity between the two stored memories, measured by the number of overlapping units, *a*, per position in the environment. To illustrate primary characteristics of these parameters we contrasted a feed forward model (*J = 0*) with two feedback models that either had orthogonal memories (*a = 0 overlapping units per position*) or memories with 67% neural overlap (*a = 12 out of 18 overlapping units per position*).

Robust rate remapping was observed in response to a change in LEC input for a wide range of parameters. Peak firing rates of simulated place fields differed substantially between the two contexts, as measured by the correlation between the vectors of peak rates for each context (feedback model, *a* = 12: *r* = 0.08, *N* = 1784; feedforward model: *r* = 0.01, *N* = 2693). The positions of model place fields were similar for both LEC contexts, as measured by a spatial correlation measure both for the feedback model with overlapping memories (*r* = 0.74±0.004; mean ± s.e.m.) and feedforward model (*r* = 0.81±0.003). Experimental measurements from the CA3 are similar to the feedback model (*r* = 0.74±0.05 [12]), while empirically measured CA1 neurons have lower spatial correlation (*r* = 0.46±0.03 [12]) likely reflecting the multiple place fields of CA1 place cells. The fact that rate remapping can be performed both by feedback and feedforward architectures is in agreement with experimental data and other modeling reports [22].

### The morph experiment

**Figure 2:**
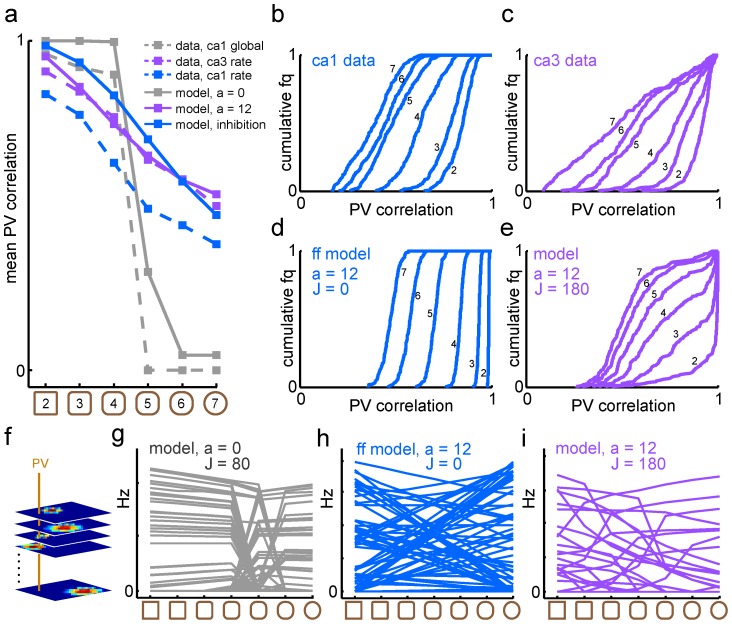
Comparison of model output to hippocampal data in the morph experiment. ***a***, Mean correlation between population vectors across the morph sequence. Experimental data in dotted lines and model output in solid lines. Blue: Data from CA1 rate remapping and feedforward model. Purple: Data from CA3 rate remapping and feedback model with overlapping memories. Gray: Data from CA1 global remapping and feedback model with orthogonal memories. Experimental data curves from [12] and [4]. ***b-e***, Cumulative distribution functions (CDF) of population vector correlations for the 6 morphed environments numbered 2-7. ***b***, for CA1 rate remapping data (Adapted from [12]). ***c***, for CA3 rate remapping data (Adapted from [12]). ***d***, for the feedforward model. ***e***, for feedback model with overlapping memories (*a* = 12). ***f***, Schematic of a population vector (PV). For each position of the environment, a PV consisting of the firing rate of each unit at that position was constructed. Each PV of the first morph shape was then correlated with the corresponding PV of the subsequent 6 morph shapes. The mean of these PV correlations are plotted in *a* for each morph shape. ***g-i***, Maximal firing rates of individual units in the model as the morph sequence progressed from square to circle. ***g***, Units in the feedback model with orthogonal units transitioned abruptly and coherently around the same point of the morph sequence. ***h***, Units in the feedforward model followed the linear change in the contextual input across the morph sequence. ***i***, Units in the feedback model with overlapping memories displayed a heterogeneous pattern of responses with both gradually transitioning units and units that transitioned abruptly at different points along the morph sequence.

If discrete episodic-like memories are stored as attractor states in a recurrent network, feedback connections should affect network responses when inputs are gradually morphed from memory A to memory B. To simulate the morph experiment [12], [13] we linearly changed the LEC input pattern from context A to context B in six steps 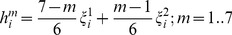
(3)For each of the seven LEC input patterns, ***h*** *^m^*, the virtual rat explored each position of the environment once, following a contiguous path. Network output depended on the strength of recurrent feedback, the amount of overlap in the stored memory patterns, and on the particular instantiation of the random memories stored in the recurrent weights. Network output qualitatively matched experimental recordings for the feedback and feedforward model parameter values (Figure 2).

Without feedback connections (*J = 0*), both average population activity (Figure 2a, solid blue line) and single unit activity (Figure 2g) transitioned smoothly from the representation of context A to context B. The distribution of population vectors in the feedforward model was narrow with the peak gradually shifting towards lower values, as indicated by almost equally spaced sigmoidal curves in the cumulative distribution function (CDF) (Figure 2d). A similar pattern was seen in the CA1 data from [12], where the peak of the distribution of population vectors gradually shifted from high correlations to lower correlations while the shape of the distribution (slope of the CDF) was preserved (Figure 2b).

For the feedback model with overlapping memories, average population activity transitioned smoothly (Figure 2a, solid purple line) while individual unit responses displayed either smooth or abrupt transition curves with a heterogeneous distribution of transition points along the morph sequence (Figure 2h). As for data from CA3 rate remapping [12] (Figure 2c), the distribution of population vector correlations gradually widened rather than merely shifting the peak towards lower correlation values, and some population vectors remained highly correlated with the initial shape even in the last morph shape (Figure 2e).

With orthogonal memories, average population activity more closely resembled data from a global remapping regime [4]. The representation abruptly switched from the initial morph shape to the final morph shape (Figure 2a, solid gray line), a behavior reflected in the abrupt and largely coherent firing rate curves of individual units (Figure 2f).

These results demonstrate that cardinal differences in the phenomenology of remapping could be explained by differences in internal network connectivity, both between hippocampal areas and within the CA3.

### Contextual attractor dynamics

**Figure 3:**
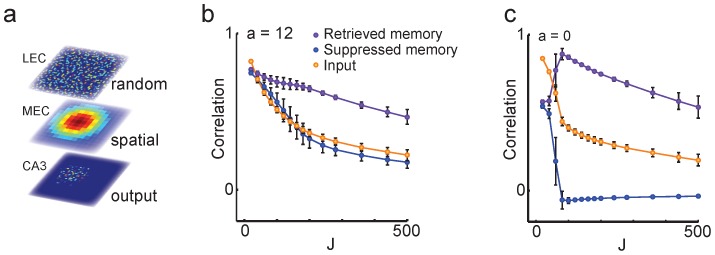
Discrete attractor dynamics for context in the feedback models. ***a***, Random LEC input was provided with the spatial MEC input to test whether model output would converge to one of the two stored patterns. ***b***, Feedback model with overlapping memories (*a* = 12). Pattern completion as a function of feedback strength, *J*, in Eq. 1. For sufficiently strong feedback, model output correlated significantly more strongly with one of the stored memory patterns (purple) than the random input pattern (yellow) or the other stored memory pattern (blue). ***c***, The pattern completion effect was even stronger for the feedback model with orthogonal memories (*a* = 0). Colors as in *b*.

We next asked whether networks with a gradual population transition curve can still have discrete attractor states for the stored memories. To this end, we performed one thousand network simulations where the network received arbitrary positional input from the MEC and random contextual input from the LEC, and measured the correlations between network input and output. Output from the feedback network with overlapping memories was significantly more correlated with one of the stored patterns (*r_retrieved_* = 0.66±0.02; mean ± s.d.) than the random input (*r_input_* = 0.38±0.02; t = 288; d.f. (degrees of freedom)  = 1998; Figure 3b). Therefore, a network can retrieve contextual memories even if it displays gradual population transitions through the morph sequence. As expected, for the network with orthogonal memories this pattern completion effect was even stronger (*r_retrieved_* = 0.88±0.04 vs *r_input_* = 0.44±0.03; t = 297; d.f. = 1998; Figure 3c). As long as MEC input was stronger than input from LEC, the influence of their relative balance (*E*) on the pattern completion results was negligible (data not shown).

### Spatial attractor dynamics

**Figure 4:**
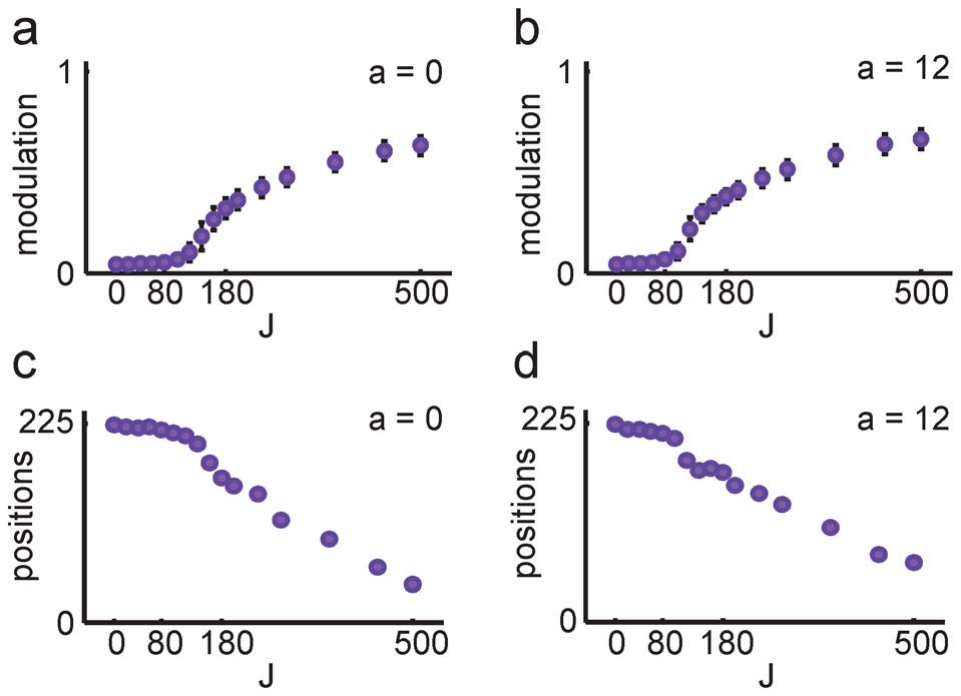
Spatial attractor dynamics in the feedback models. ***a***, To test whether the model exhibited spatial attractor dynamics, random LEC input was provided in the absence of MEC input. ***b-c***, Spatial modulation in the absence of MEC input for the feedback model with orthogonal memories (*a* = 0) in ***b***, and overlapping memories (*a* = 12) in ***c***. The model was simulated with 1000 random LEC input patterns and no MEC input over a range of feedback strengths (*J*). The output showed significant spatial tuning for large *J*. Mean of 1000 simulations is shown for each value of *J*. Error bars denote STD. ***c-d***, Spatial stability, measured as the number of positions the network converged on in the absence of MEC input, decreased as a function of feedback strength for both feedback models.

Network connectivity was structured to support both spatial and contextual attractor dynamics. The spatial component served to confine activity to a single activity bump of CA3 units ensuring the expression of single place fields. MEC input determined the position of the CA3 bump and provided sufficient spatial stability to move CA3 activity along the simulated path. To investigate how spatial stability depended on MEC input, we performed one thousand network simulations with random LEC input but no MEC input. Even without MEC input network activity converged to a single bump of activity for random LEC input. The spatial tuning of the network activity increased with recurrent strength, both for the network with orthogonal memories (Figure 4a) and overlapping memories (Figure 4b), but at the expense of spatial stability, a familiar issue for continuous attractor networks [15], [23]. Spatial stability, measured as the number of stable positions that could be retrieved for the 1000 random LEC input patterns, decayed gracefully with increasing recurrent weight strength (Figures 4c and 4d).

### Morph transition dynamics

Why do the contextual attractor dynamics of the network with overlapping memories not show up as abrupt transitions across the morph procedure? One possibility is that a hysteretic effect biases the representation towards the initial context, so that activity never completely escapes from the attractor of context A. Whereas place cell firing rate in the dentate gyrus is a direct function of the current morph stage, the firing rate of single CA3 neurons depends on the direction of morphing [13]. Although this kind of hysteresis could be an effect of short term plasticity [24], it is also a generic property of attractor networks [25].

**Figure 5:**
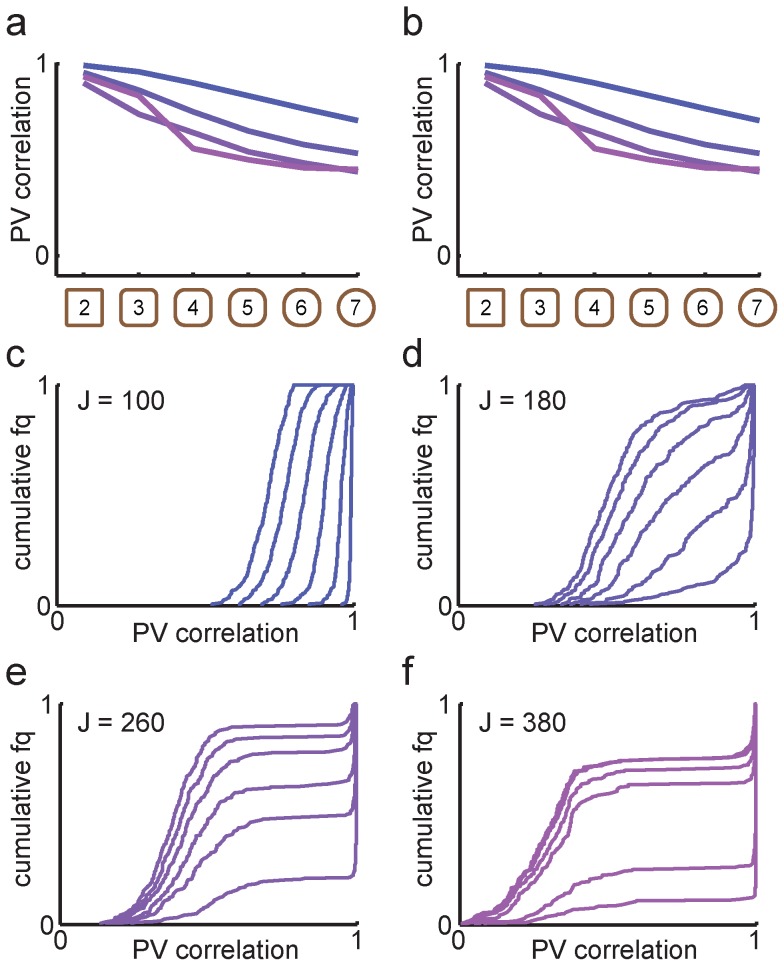
Transition curves in the model with overlapping memories (*a* = 12) were not shaped by hysteresis. ***a***, Mean population vector correlation transition curves for different feedback strengths. Increasing *J* was associated with stronger pattern separation and sharper transition curves. From blue to purple, *J* = (100, 180, 260, 380). ***b***, To test history dependence of the transition curves, new simulations where network activity was reset between every morph shape were performed. The mean transition curves were not affected by the manipulation. ***c-f***, The CDF curves corresponding to the mean population vector correlations in ***a*** qualitatively changed as *J* increased. For sufficiently strong feedback, sharper transitions could be seen as an asymmetry in how separated the CDF curves were.

The strength of recurrent feedback substantially affected the population response through the morph sequence (Figure 5). As feedback strength was increased, the representation of the two end shapes became less correlated (Figure 5a), and the CDF curves showed wider distributions of population vector correlations (Figures 5c–f).

To explore the effect of hysteresis on the network activity in the CA3 model, we first simulated a version of the scrambled morph experiment [12], in which network activity was reset to zero between each morph stage. For the network with overlapping memories, resetting network activity only marginally affected population vector correlations (Figures 5a and 5b). Both the graded transitions and the shape of the CDFs were maintained without hysteresis. This is consistent with the original experimental observation that scrambled intermediate shapes produced the same population response patterns as the sequential morph [12], and suggests that gradual population transitions with wide population vector distributions is not dependent on hysteresis or plasticity but rather a signature of the stored memory patterns.

**Figure 6:**
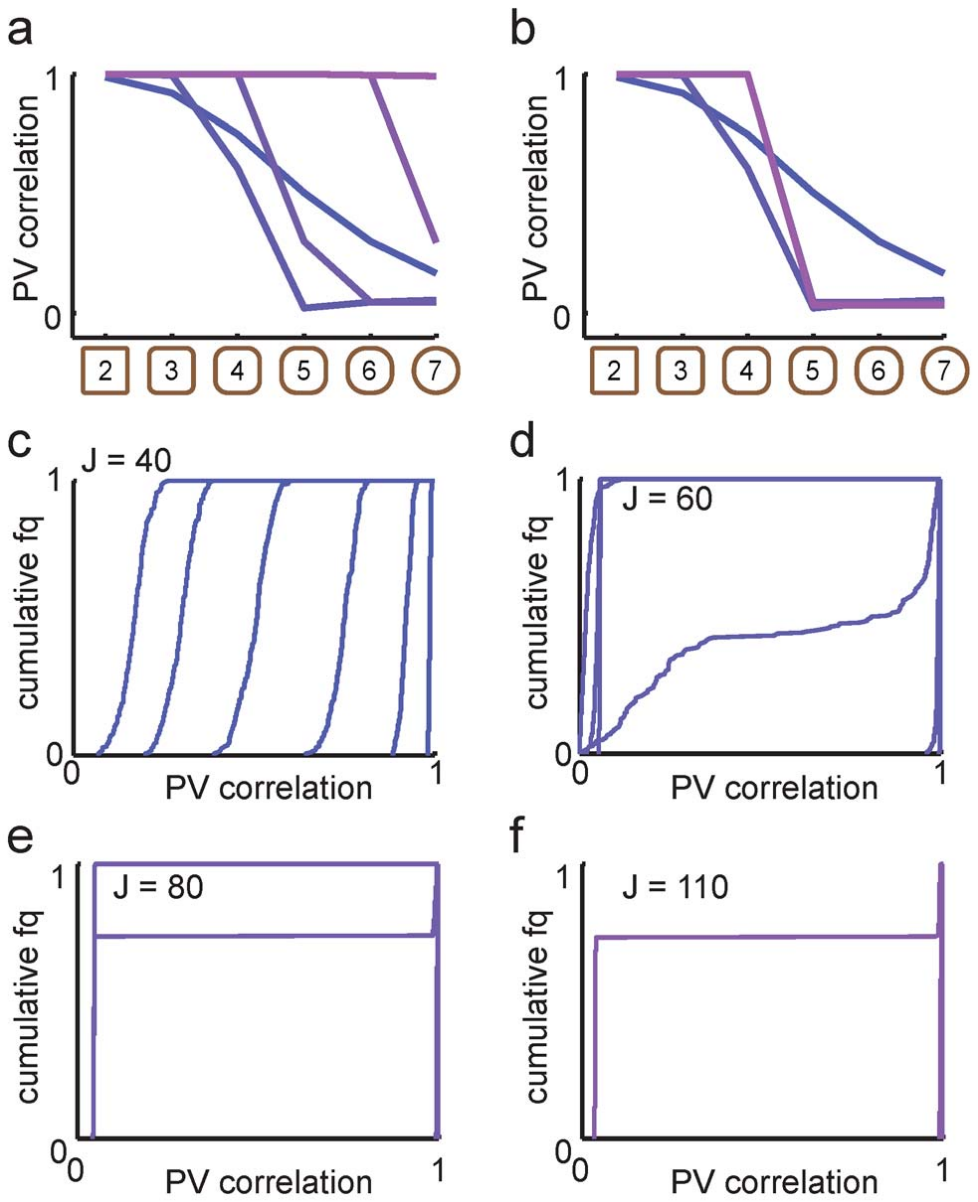
Population transitions and hysteresis in networks with orthogonal memories (*a* = 0). ***a***, Mean population vector correlation transition curves for different feedback strengths. Increasing *J* lead to sharper transition curves with strong pattern separation and progressively stronger hysteresis. From blue to purple, *J* = (40, 60, 80, 110). ***b***, When network activity was reset between each morph shape, the hysteretic effect disappeared and the network transitioned at the midpoint of the morph sequence for sufficiently strong feedback. ***c-f*** *,* The cumulative population vector correlation curves changed as a function of *J*.

For the network with orthogonal memories, average population transitions were abrupt even for small values of J (Figure 6a). The CDF curves (Figure 6d–f) also markedly differed from the case with overlapping memories (Figure 5d–f), and wide distributions of population vector correlations were not observed for any setting of the parameters. Resetting network activity between shapes had a profound effect on population activity in the network with orthogonal memories (Figure 6b), indicating a strong effect of hysteresis due to non-linear network dynamics. The effect of network-wide hysteresis increased with feedback strength until the network ultimately remained trapped in the initial representation (Figure 6a, pink line). This is reminiscent of strong population level hysteresis observed for both hippocampal and entorhinal representations in a global remapping regime (Supplementary figure 11a in [26]).

**Figure 7:**
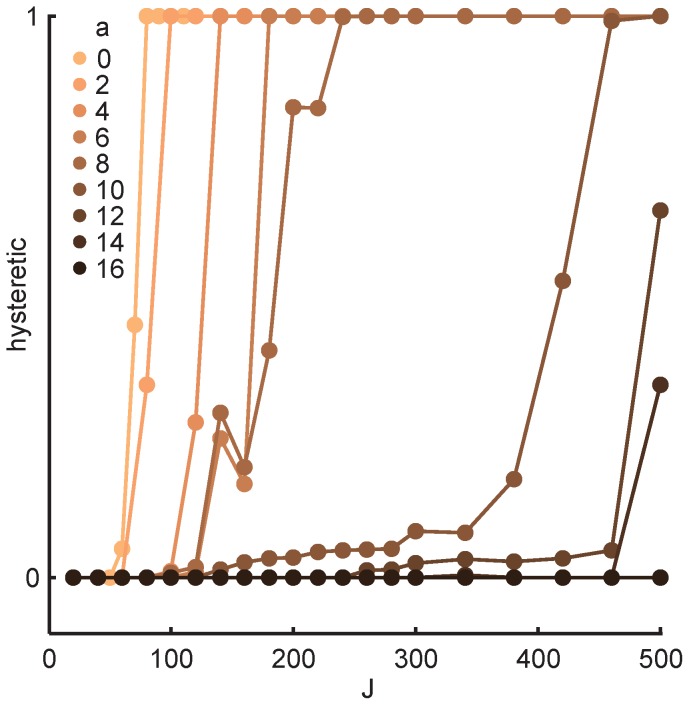
Single unit hysteresis. A hysteretic unit was defined as a unit with more than 10% deviation in firing rate between morphing directions in at least one morph shape. The figure shows the proportion of hysteretic units as a function of feedback strength (x-axis) and memory overlap (colors). Darker colors indicate more overlapping units in the two stored memories.

To assess the effects of hysteresis on the single unit level, we simulated the reverse morph sequence and found that the subset of units with firing rates that depended on the direction of morphing increased with the strength of feedback and decreased with the amount of overlap in the stored representations of context (Figure 7). The fraction of hysteretic units also varied with the particular random memories stored in the network (data not shown). In other words, a small number of hysteretic units were observed even in networks whose mean population response transitioned gradually across the morph sequence. The prevalence and inter-subject variability of hysteretic units in rate remapping experiments still awaits quantification, but substantial variability in the effect of hysteresis has already been documented between animals in the global remapping regime [4], [26].

### Signature of spatio-contextual attractor dynamics

**Figure 8:**
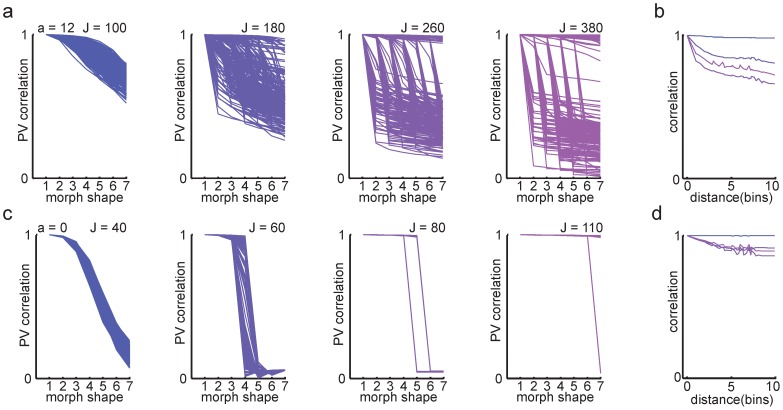
Signature of local context-dependent attractor dynamics for overlapping and orthogonal memories. **a**, Overlapping memories. Correlation with square shape for all 225 population vectors across the morph sequence. Parameters and colors as in Figure 5. Stronger *J* lead to incoherent transition points between population vectors. ***b***, Spatial dependence of population transitions. Transition curves for population vectors representing nearby positions had more similar transition points than population vectors representing distant positions, leading to a spatial profile. The spatial profile was not present for the feedforward network (blue line). ***c***, Orthogonal memories. Population vector correlation curves for networks with orthogonal memories were largely coherent. ***d***, A weaker spatial profile than in ***b*** was found for the correlations between transition curves of population vector correlations for orthogonal memories.

To understand how correlations in the stored memories induce gradual population transitions, we plotted the transition curve for each position-dependent population vector in the feedback model (Figure 8a). Rather than having one coherent context attractor, the network displayed multiple position-dependent attractors that transitioned at different points along the morph sequence. The average of many individual abrupt transitions appears gradual on the population level (Figure 5a). This explains why single cell responses can be abrupt while the population average is smooth, and predicts a spatial signature in transition dynamics for the population (Figure 8b). Place cells with place fields close to each other in space should have a tendency to transition at similar points along the morph sequence. However, some units may display smooth transitions if they participate to similar degrees in both memories. In a network with orthogonal memory representations, population vector correlation profiles were much more stereotyped (Figure 8c), and the spatial profile weaker but still present for strong feedback (Figure 8d). The spatial profile of population vector correlations is a result of local cell assemblies for contextual features embedded in a spatial attractor manifold.

## Discussion

Recent evidence supports a behaviorally relevant role for firing rate modulation in context-dependent memory [8], but whether rate remapping is governed by attractor dynamics is unclear [5], [27]. We analyzed the possibility that CA3 memories consist of local attractor states embedded in a continuous spatial map [12]. Implementing this architecture in a network model was sufficient to reproduce the main features of CA3 rate remapping on both the single neuron and network levels in a way that was consistent with episodic-like memory. The results support the view that place cells process multimodal information through attractor dynamics.

### Attractor dynamics

The key ingredient in the CA3 model was the discrete attractors for contextual features stored locally within a broader spatial attractor manifold in the recurrent weight matrix. The spatial component of the synaptic feedback confined activity to place cells with similar positional preferences and ensured that the position with the strongest collective input was activated. Input was dominated by spatial activity from MEC, implying that a relatively weak signal for context is sufficient to elicit rate remapping. However, spatial activity patterns were formed even without MEC input, showing that any source of spatial information suffices to produce place field responses. This spatial pattern formation mechanism depends on dynamics of continuous attractors [23], [28]–[30]. The required intrinsic spatial structure is consistent with the empirical observations that CA3 place cells tend to have unimodal place fields [13] (but see [31], [32]) and that place cells preserve their spatial firing selectivity in the absence of either self-motion cues [33] or visual information [34]–[36].

The discrete component of the synaptic weights forced CA3 activity towards one of the stored context memories in a process of pattern completion, with the LEC input serving to bias which of the two stored patterns were expressed. The contextual pattern formation mechanism required LEC input to convey rate changes in response to manipulations of external context, in agreement with the finding that rate remapping is impaired in animals with neurotoxic LEC lesions [37].

### Global remapping

Global remapping is thought to involve independent linear transformations of different grid cell modules [26], [38], [39], but whether the transition is a coordinated effort or primarily driven by either MEC or hippocampus is not known. Although the role of the MEC in global remapping was not explicitly analyzed in the present study, model simulations using orthogonal memory patterns produced sharp and coherent transition dynamics in response to relatively weak, linear changes in LEC input. Whereas rate remapping might be an expression of switching between local attractors within a common spatial map, global remapping might express a switch between spatial maps represented by unrelated neural charts [30], [40]. The defining difference between rate remapping and global remapping might therefore be the structure of the CA3 feedback connections as much as the difference in input, an interpretation which is commensurate with a recent analysis of the impact of storing correlated memories in continuous attractor networks [41].

### Hysteresis

In the empirical morph experiment, strong hysteresis led the authors to suspect the presence of attractor dynamics [12]. One major difficulty in isolating the role of hysteresis in these experiments is that long-time spatial averaging of population vector correlations is a coarse measure of true network dynamics. In the experiments, the animal is removed from the arena for several minutes before being placed back into the next morph shape. During this time, contextually related activity must be retained or facilitated by the animal in order for a hysteretic effect to contribute to the network dynamics. Hippocampal neurons are reported to maintain their place-specific firing in the absence of landmarks or visual cues over similar extended periods of time [34], [35], [42]. The role of synaptic plasticity in such working memory remains unknown but the phenomenon is likely to be supported by mechanisms like synaptic facilitation [43] or gain modulation [15].

Another observation from the experimental study was that the representation of the square and circular arenas were more similar after experiencing the morph sequence than at pre-training. Again, it is difficult to determine whether this decreased pattern separation (a) is due to hysteresis in a strict dynamical systems sense, (b) stems from a network whose response to new stimuli is slow but ultimately independent of starting point, or (c) results from a network that undergoes synaptic plasticity through the morph sequence. From the perspective of the model, the experimental observation can either be explained by a modulation of the balance between entorhinal input and hippocampal feedback or as a plasticity-dependent increase in the overlap between the hippocampal representations of square and circle. Although it is not clear why only the CA3 would be prone to such representational malleability, synaptic plasticity is also implied by the change in CA3 response to the morph sequence over multiple exposures [12]. To accurately distinguish between these possibilities however, analysis of network dynamics just before and shortly after morph shape transitions are required. Such analyses are just becoming feasible, as large-scale neural recording technology is reaching maturity (e.g. [44]) and can be combined with new experimental designs that allow for investigating remapping at the appropriate time scale [45].

### Functions of CA3 attractor dynamics

The present model of CA3 rate remapping relies on the combination of continuous and discrete attractor dynamics, and extends previous models for how both 'what' and 'where' information can be stored in an attractor network [15], [16], [46]. An intriguing consequence of the proposed architecture is that it allows local modifications to a pre-existing representation of an episode or spatial map without affecting other neurons coding for distant positions. This flexibility to locally update the place field map were experimentally observed in rats when a novel shortcut was introduced in a familiar maze [47], [48], when objects were moved within a familiar arena [49]-[51], and when the goal location was relocated within a Morris water maze task [52]. To ensure spatial stability under conditions where external input is temporarily unavailable, additional stabilizing mechanisms may be important [15], [53], [54]. However, rather than avoiding drift, spatially dependent recurrent connections in CA3 could serve to encode paths and topological relationships between contextual features in the environment, consistent with the view that hippocampal neurons encode "phase sequences" [5], [55] and are involved in path planning [56], [57].

### Rate remapping in the dentate gyrus

Rate remapping in the dentate gyrus has been shown to be different from that in CA3 [13]. The dentate gyrus is generally believed to perform a pattern separation of inputs to CA3 [11], consistent with models showing that rate remapping in the dentate gyrus can be explained in terms of inhibition-driven pattern separation of inputs from the entorhinal cortex [22] or a synaptic gating mechanism [58], [59]. In principle, attractor dynamics could also be part of the disynaptic connections of the Mossy-Hilar system [60], and feedback connections from the CA3 could influence dentate dynamics. Whether the dentate and CA3 operate as a unit or perform separable functions remains to be determined. The present model predicts that CA3 rate remapping can still be observed after dentate lesions, consistent with empirical data showing that spatial selectivity of CA3 place fields is preserved after dentate lesions [61].

### Model predictions

How can we test whether attractor dynamics impact the discharge patterns of CA3 place cells? The model makes several predictions about place cell activity. First, we found that the transition curves of nearby population vectors are more similar than the transition curves of distant population vectors (Figure 8). This spatial dependence in population activity transitions can be measured from large-scale CA3 recordings in a rate remapping paradigm. Furthermore, the signature should depend on active recurrent collaterals, and require synaptic plasticity to form. Second, single cell hysteresis is expected even in the absence of synaptic plasticity, and should also be dependent on active CA3 feedback. Third, CA3 rate remapping should be independent of the dentate gyrus provided that memories have already been formed. Fourth, while rate remapping is dependent on LEC input, spatial preference is expected to be flexible with regard to the functional source of spatial information because it is encoded in the recurrent connections. Finally, attractor dynamics are sensitive to the balance between entorhinal input and recurrent feedback. Molecular techniques for manipulating the strength of population activity could provide means to test this prediction.

### Conclusions

This study shows that attractor dynamics could provide a mechanism for connecting discrete memories with a representation of space in a way that is consistent both with neurophysiology and current theory for episodic-like memory [62]. The phenomenon of rate remapping is reminiscent of 'gain fields' in sensorimotor transformations, a multiplicative modulation of tuned responses, as in brain areas responsible for eye and hand position in the parietal reach region [63], [64]. Gain fields typically represent two or more continuous variables whereas rate remapping involves more discrete patterns of activity, but both phenomena could share similar mechanisms on network, neural, and synaptic levels [65]. Understanding rate remapping might therefore be important to our understanding of a much wider range of brain areas [66], [67].

## Methods

### Simulations

All simulations were based on a 75×75 cm^2^ recording arena discretized into position bins of 5×5 cm^2^, similar to [12]. Each of the 225 positions had eighteen hippocampal units associated with it, for a total of 4050 hippocampal units. In a simulated contiguous path taken by the rat every position of each context was visited once, long enough for the network activity (Eq 1) to converge. Network activity was not reset between positions or context shapes except when explicitly mentioned. Periodic boundary conditions were imposed on the environment, which had the topology of a torus. All network simulations were run on Matlab 2012b (Mathworks) using a forward Euler integration scheme. Convergence was assumed when the mean difference between unit activities was less than 3×10^−5^ from one time step to the next. For the standard parameters, simulations reached this convergence criterion in 239±100 (mean ± s.d.) iterations.

### Network dynamics

The activity of the hippocampal units was governed by Eq 1. All inputs were excitatory, and inhibition was provided both through a subtractive term in the recurrent weights and a divisive term in the activation function (Eq 3).

For the feed forward network (*J* = 0) in Figure 2, a feed forward inhibition was added to the network to compensate for the missing subtractive inhibition in the recurrent weights which increases sparsity (pattern separation). The dynamics were thus governed by the following equation: 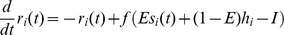
where *I* = 0.8 and the other parameters were as for Eq 1.

In the simulations, an activity bump contained 210±10 (mean ± s.d.) active units per position for the model with overlapping memories (*a* = 12), and 293±13 active units per position for the model with orthogonal memories (a = 0).

### Recurrent weights

Recurrent hippocampal activity was provided through the connectivity matrix, *w*_,_ in which a single shot Hebbian learning rule stored the context representations (Eq 2). Two pattern vectors, ***ξ*** *^1^* and ***ξ*** *^2^*, of CA3 network activity were generated to represent the square and circular contexts respectively (*M = 2*). The number of active (non-zero) units was the same for both patterns and all positions. The number of overlapping units per position in ***ξ*** *^1^* and ***ξ*** *^2^* was denoted by the parameter *a* and ranged from 0 to 18 in steps of two units. The level of activity for active units was drawn from a uniform probability distribution. For networks with orthogonal memories, *a* = 0, nine units were active in each pattern and pattern vectors satisfied ***ξ*** *^m^*. ***ξ*** *^n^* = 0 for *m* ≠ *n*, where bold denotes vector notation.

### Analysis

Rate maps were constructed for each hippocampal unit by calculating firing rate as a function of the 15×15 position bins for each environment. Based on these rate maps, population vectors, population vector correlations, cumulative frequency plots of population vector correlations, spatial correlations, and rate overlap measures were calculated as in [12].

#### Spatial modulation index

The spatial modulation index was taken to be the fraction of the total activity that was within a 5 by 5 bins square around the circular mean of the activity in the two-dimensional network.

#### Measure for stable positions

The position read out by the hippocampal network was defined as the circular mean of the hippocampal network activity. The number of stable positions was measured as the number of unique positions that the network activity converged on after 1000 simulations with random LEC input patterns. The maximal number of positions was 225.

#### Measure of context correlation between the output pattern and stored patterns

The contextual correlation between the hippocampal output, ***r***, and a contextual pattern for a given position (Figure 3) was calculated as *corr(* ***r*** *,* ***h*** *·* ***s*** *)*; where ***h*** is an LEC input pattern or one of the stored patterns, ***ξ*** *^1^* and ***ξ*** *^2^.* ***s*** is the MEC input pattern at the current position thresholded at 0.3 to achieve spatial confinement.

#### Spatial profile of population vector correlations

For each morph shape, the population vector correlation for each position was calculated and plotted in Figures 8a and 8c. The spatial correlation profiles in Figures 8b and 8d were calculated as the correlation between the population vector correlation transition curves at different positions (Figures 8a and 8c) and plotted as a function of the spatial distance between their positions.

#### Measure of hysteresis

Hysteresis was quantified by comparing the rate curve arising from morphing context *A* into context *B* with that arising from morphing context *B* into context *A*. A unit was counted as hysteretic if its firing rates in the two morph directions differed by more than 10% of the difference between its overall maximum and minimum rates, in at least one morph shape.
